# Pathomics and single-cell analysis of papillary thyroid carcinoma reveal the pro-metastatic influence of cancer-associated fibroblasts

**DOI:** 10.1186/s12885-024-12459-4

**Published:** 2024-06-10

**Authors:** Yixian Wang, Xin Li, Qingwei Gang, Yinde Huang, Mingyu Liu, Han Zhang, Shikai Shen, Yao Qi, Jian Zhang

**Affiliations:** 1 Department of Vascular and Thyroid Surgery, The First Hospital, China Medical University, Shenyang, Liaoning 110001 China; 2 Department of Head and Neck Surgery, Liaoning Cancer Hospital & Institute, Shenyang, Liaoning 110042 China; 3 Department of Breast and Thyroid Surgery, Chongqing General Hospital, Chongqing, 401147 China

**Keywords:** Papillary thyroid carcinoma, Cancer-associated fibroblasts, Pathomics, Single-cell RNA sequencing, Deep learning

## Abstract

**Background:**

Papillary thyroid carcinoma (PTC) is globally prevalent and associated with an increased risk of lymph node metastasis (LNM). The role of cancer-associated fibroblasts (CAFs) in PTC remains unclear.

**Methods:**

We collected postoperative pathological hematoxylin–eosin (HE) slides from 984 included patients with PTC to analyze the density of CAF infiltration at the invasive front of the tumor using QuPath software. The relationship between CAF density and LNM was assessed. Single-cell RNA sequencing (scRNA-seq) data from GSE193581 and GSE184362 datasets were integrated to analyze CAF infiltration in PTC. A comprehensive suite of in vitro experiments, encompassing EdU labeling, wound scratch assays, Transwell assays, and flow cytometry, were conducted to elucidate the regulatory role of CD36^+^CAF in two PTC cell lines, TPC1 and K1.

**Results:**

A significant correlation was observed between high fibrosis density at the invasive front of the tumor and LNM. Analysis of scRNA-seq data revealed metastasis-associated myoCAFs with robust intercellular interactions. A diagnostic model based on metastasis-associated myoCAF genes was established and refined through deep learning methods. CD36 positive expression in CAFs can significantly promote the proliferation, migration, and invasion abilities of PTC cells, while inhibiting the apoptosis of PTC cells.

**Conclusion:**

This study addresses the significant issue of LNM risk in PTC. Analysis of postoperative HE pathological slides from a substantial patient cohort reveals a notable association between high fibrosis density at the invasive front of the tumor and LNM. Integration of scRNA-seq data comprehensively analyzes CAF infiltration in PTC, identifying metastasis-associated myoCAFs with strong intercellular interactions. In vitro experimental results indicate that CD36 positive expression in CAFs plays a promoting role in the progression of PTC. Overall, these findings provide crucial insights into the function of CAF subset in PTC metastasis.

**Supplementary Information:**

The online version contains supplementary material available at 10.1186/s12885-024-12459-4.

## Introduction

Thyroid cancer (THCA) stands as the most prevalent malignant tumor within the endocrine system [1]. Among the various pathological types of THCA, papillary thyroid carcinoma (PTC) emerges as the most widespread [2]. Notably, the incidence of PTC has exhibited a rapid increase on a global scale in recent years [3]. The presence of neck lymph node metastasis (LNM) in PTC is acknowledged as a risk factor associated with local recurrence, distant metastasis, and diminished survival rates [4, 5]. Therefore, unraveling the molecular mechanisms governing the invasion and metastasis of PTC assumes paramount significance.

Tumor tissues encompass both tumor parenchyma and stroma. The infiltration and metastasis of tumor cells intricately link to synergistic interactions with stromal components, facilitating mutual nourishment and propelling further cancer progression [6]. Within the tumor microenvironment (TME), stromal cells wield regulatory control over diverse biological behaviors of tumor cells, including proliferation, apoptosis, migration, and invasion. This orchestration facilitates tumor development, while tumor cells reciprocally reshape the TME by modulating stromal cells, ultimately enhancing angiogenesis and metastasis [7, 8]. A pivotal player in this scenario is cancer-associated fibroblasts (CAFs), constituting a major stromal cell population in the TME. They originate from normal fibroblasts at the tumor site or undergo transformation from circulating bone marrow-derived mesenchymal stem cells [9]. CAFs comprise distinct subpopulations with varied functions in tumors [10]. Specific CAF subgroups exert positive influences on diverse facets of tumor growth, encompassing cancer cell survival, proliferation, vascularization, and extracellular matrix (ECM) remodeling, thereby impacting metastasis [9]. CAFs accelerate tumor cell growth and metastasis, directly or indirectly affecting the progression of various cancers, including lung cancer [11], breast cancer [12], colorectal cancer [13], pancreatic cancer [14], and gastric cancer [15]. By secreting growth factors, CAFs stimulate cancer cell proliferation and invasion [16], shape the innate and adaptive immune cell responses to cancer cells via cytokine secretion [17], and transport molecules to cancer cells via extracellular vesicles, promoting their invasiveness [18]. The functionality of CAFs critically affects treatment responses and resistance development [19], positioning CAFs as potential cancer therapy targets.

**Fig. 1:**
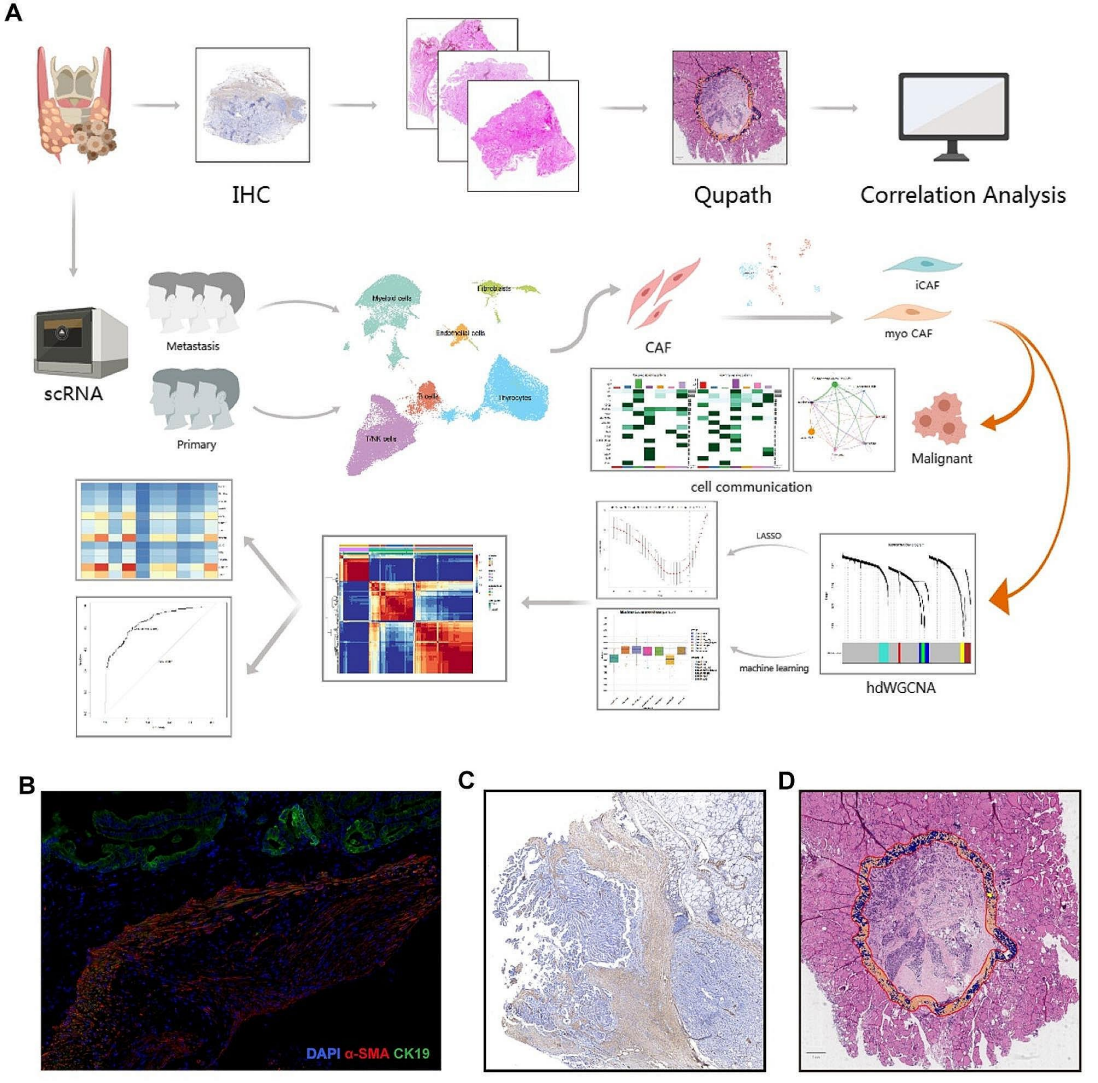
Process flowchart and hematoxylin–eosin slide annotation of tumor invasive front with cancer-associated fibroblasts (CAFs). (**A**) Process flowchart. (**B**) Immunofluorescent double staining results for α-SMA and CK19. (**C**) Smooth muscle actin-alpha (α-SMA) immunohistochemical staining. (**D**) Schematic representation of Qupath delineation of tumor invasive front and CAF annotation. The orange areas represent fibroblasts and tissues, while the blue areas represent tumor cells

Despite the established significance of CAFs in various cancers, their precise role in PTC remains incompletely elucidated. Some studies have hinted at a correlation between CAFs and PTC development [20, 21]. However, the specific molecular mechanisms by which CAFs foster the occurrence and progression of PTC remain unexplored. Digital pathology, integral to modern clinical practice [22], has evolved with the advent of whole slide imaging (WSI), enabling the imaging of complete glass slides with high-resolution storage. In this study, we utilized a substantial dataset of pathology WSI from clinical patients to analyze the pathological dimension of the correlation between tumor fibrosis level and LNM. With the advancements of single-cell RNA sequencing (scRNA-seq) technology, in-depth research on the role of CAFs in cancer has been extensively studied using scRNA-seq data analysis [23, 24]. We utilized publicly available scRNA-seq PTC data to characterize the features of CAFs within PTC and describe their interactions with other cell types. The study flowchart is illustrated in Fig. 1A.

## Materials and methods

### Data acquisition

#### Clinical patient data acquisition

Patients with PTC at the First Affiliated Hospital of China Medical University in 2017 were selected. Inclusion criteria comprised an initial PTC diagnosis post-surgery, an absence of prior radiotherapy or chemotherapy, and complete clinical data. Exclusion criteria included non-PTC pathology post-surgery, preoperative treatment, incomplete clinical data, and coexisting malignancies. Following stringent criteria-based selection, a total of 984 patients were enrolled. Postoperative hematoxylin–eosin (HE) slides were systematically collected for panoramic scanning, and patient data, including age, sex, anatomic site, focus type, T grade, N grade, M grade, clinical stage, coexistence with Hashimoto’s disease, and extrathyroidal extension, were meticulously recorded.

#### scRNA-seq data

Single-cell transcriptome data from GSE193581 and GSE184362 were meticulously downloaded from the Gene Expression Omnibus (GEO) database (http://www.ncbi.nlm.nih.gov/geo/). The analysis included five PTC lesions with LNM and one PTC lesion without metastasis from the GSE193581 dataset, as well as four PTC lesions with LNM and three PTC lesions without metastasis from the GSE184362 dataset. Harmony, a bioinformatics tool, was proficiently employed to seamlessly integrate and harmonize the datasets.

#### Bulk RNA-seq data

The THCA expression and clinical data were sourced from The Cancer Genome Atlas (TCGA) data portal (https://tcga-data.nci.nih.gov/tcga/) and the UCSC cancer browser (https://genome-cancer.ucsc.edu), respectively. The TCGA THCA dataset comprised 510 PTC samples and 58 non-cancer samples. As an external validation cohort, the GSE33630 dataset was systematically utilized, downloaded from the GEO database (https://www.ncbi.nlm.nih.gov/geo/). Following the exclusion of patients with missing clinical information, a comprehensive total of 49 patients with PTC were included in the analysis. Fragments per Kilobase Million was employed as the normalized value for bulk RNA-seq analysis.

### Immunohistochemical staining

Immunohistochemical staining was methodically conducted using a specialized immunohistochemical kit and 3,3’-diaminobenzidine substrate kit, meticulously following the manufacturer’s protocol. Tissue sections were incubated overnight at 4 °C with primary antibodies against smooth muscle alpha-actin (α-SMA) (Abcam, Cambridge, MA; dilution 1:100).

### Immunofluorescence staining

Double immunofluorescent staining involved deparaffinization, rehydration, and heat-induced epitope retrieval of tissue sections. Subsequent to permeabilization and blocking, sections were incubated overnight at 4 °C with CK19 (Abcam, #ab76539) and α-SMA (Abcam, #ab7817) antibodies. Following thorough washing, fluorochrome-conjugated secondary antibodies were used for the simultaneous visualization of multiple antigens. Nuclei were counterstained with 4’,6-diamidino-2-phenylindole (DAPI), and sections were mounted with an anti-fade medium.

### Evaluation of fibrosis level in HE pathological tissue sections

An open-source software for digital pathology image analysis, QuPath, was used to meticulously assess fibrosis levels in HE pathological tissue sections. Within QuPath, tumor boundaries were delineated on each HE-stained slide, and an area within 1000 μm from this boundary was designated as the invasive front of the tumor. A pixel classifier was developed to identify fibroblasts and fibrotic regions at the invasive front of the tumor using machine learning techniques. The ratio of the identified fibrotic area to the total area of the tumor invasive front yielded the fibrosis density for each HE slide. All HE slides were subjected to independent completion and verification by three pathology experts (Supplementary Table 1).

### Preprocessing and annotation of scRNA-seq data

We obtained single-cell count matrices by downloading data from GSE193581 and GSE184362. The Seurat package, a comprehensive toolkit, was used to analyze gene expression matrices for each sample. To eliminate doublets, DoubletFinder was applied. Each cell was subjected to quality control using specific criteria, including a minimum gene count of 500, a maximum gene count of 5000, and a mitochondrial proportion below 10%. We conducted principal component analysis using the most variable genes, and the top 20 principal components were utilized for Louvain clustering. Dimensionality reduction was conducted using UMAP (https://umap-learn.readthedocs.io/en/latest/).

We identified and annotated cell types based on specific gene markers [25]: TG, EPCAM, KRT18, and KRT19 for thyrocytes; CD3D, CD3E, CD3G, and CD247 for T and natural killer (NK) cells; LYZ, S100A8, S100A9, and CD14 for myeloid cells; CD79A, CD79B, IGHM, and IGHD for B cells; COL1A1, COL1A2, COL3A1, and ACTA2 for fibroblasts; PECAM1, CD34, CDH5, and VWF for endothelial cells.

### Cell communication analysis

For the comprehensive inference and quantitative characterization of cell communication networks, the “CellChat” R package, encompassing ligand, receptors, and their interactions, was employed. The analysis was conducted using the secreted signaling pathway, and the reference human ligand-receptor database, CellChatDB, was utilized to evaluate human intercellular communication. Interactions between different cell types were evaluated, with pathways containing fewer than 10 cells filtered out.

### Pseudo-time trajectory analysis

Pseudo-time analysis was systematically conducted using the Monocle2 package with default parameters. This advanced analysis included machine learning to simulate temporal progression dynamics based on the expression patterns of crucial genes. Genes exhibiting significant variations in intercellular gene expression were selected, and their expression profiles were downsampled to construct a minimum spanning tree (MST) [23]. Subsequently, we employed the MST to delineate the differentiation trajectory of cells sharing similar transcriptional characteristics along the longest path.

### High dimensional weighted gene co-expression network analysis (hdWGCNA)

For the comprehensive co-expression analysis of scRNA-seq data, the hdWGCNA R package (v0.1.1.9002) was employed (https://github.com/smorabit/hdWGCNA) [26]. To establish a co-expression gene network with a signed scale-free nature, soft-threshold parameters of β = 4 and a scale-free R2 = 0.9 were chosen to ensure network robustness.

### Gene ontology (GO) analysis and protein-protein interaction (PPI) network construction

GO enrichment analysis was conducted in the GO database (http://www.geneontology.org/). The PPI network was analyzed using the STRING protein interaction database (http://string-db.org).

### Construction and validation of cancer-associated myofibroblast (myoCAF)-related gene signature

The top 100 highly expressed genes from the brown module were selected. Furthermore, univariable Cox regression analysis was applied to identify myoCAF-related genes associated with metastasis. A gene signature was developed using least absolute shrinkage and selection operator (LASSO) penalized Cox regression analysis with the “glmnet Version 4.1.4” package in R. Thirteen myoCAF-related genes were identified, forming the gene signature.

We constructed receiver operating characteristic (ROC) curves using the R package “ROCR Version 1.0.11” to evaluate the diagnostic performance of the myoCAF-related gene diagnosis signature.

### Machine learning analysis

Seven machine learning methods, including weighted k-nearest neighbor classifier (kknn), linear discriminant analysis, logistic regression, Naïve Bayes, Random Forest regression, Decision Tree, and support vector machine (SVM), were employed to search for the most fitting formula. R packages ‘kknn’, ‘MASS’, ‘glmnet’, ‘Ranger’, and ‘rpart’ were used in this process.

### Identification of PTC subtypes

Using myoCAF-related genes associated with metastasis, non-negative matrix factorization (NMF) clustering was conducted. NMF clustering is commonly used to delineate molecular subtypes in cancer [27]. We used the NMF R package for unsupervised NMF clustering on the metadata set, and the ideal cluster number was determined based on the co-occurrence correlation coefficient K value.

### Immune cell infiltrate analysis

The ‘microenvironment cell population count (MCP-counter)’ approach with the R package MCPcounter was employed for immune infiltration assessment [28]. This method quantifies the absolute abundance of 8 immune cell types and 2 stromal cell populations based on transcriptome data.

### Cell culture

After rinsing the LNM-PTC specimens with D-Hanks solution, cut them into tissue blocks of 1 × 1 × 1 mm in size. Fetal bovine serum (FBS) is used to soak the bottom of T25 culture flasks, and then the tissue blocks are evenly spread out and placed in an incubator for pre-attachment. After pre-attachment, a specialized culture medium is added to soak the tissue blocks. This specialized culture medium contains DMEM/F12 medium with 5% FBS, 100 units/ml penicillin, 100ug/ml streptomycin, 5ug/ml insulin, 1ug/ml dexamethasone, and 5ng/ml epidermal growth factor. The medium should be changed daily for the first 3 days, then every 3–4 days afterwards, for a total culture period of 26–30 days. Discard the culture medium and tissue blocks, and proceed with the subculturing process according to the cell passage protocol.

TPC1 and K1 cells, acquired from Wuhan Puno Sai Life Technology Co., Ltd. in Wuhan, China, were grown in 1640 medium enriched with 10% FBS.

### Establishment of CD36 knockdown CAF

CD36 knockdown CAF were created via lentivirus transfection, while control cells received negative control lentivirus (NC). Verification of successful CD36 depletion was performed through western blotting. The specific CD36 shRNA sequence used was 5′-CCGGCGGATCTGAAATCGACCTTAACTCGAGTTAAGGTCGATTTCAGATCCGTTTTTG-3′. These CD36 shRNA and negative control vectors were introduced into CAF using Lipofectamine 3000 (Invitrogen, USA).

### Western blot

Western blot experiments utilized a CD36-specific antibody (ab133625, Abcam, Cambridge, UK) to probe for protein levels. The detected protein bands were visualized using an ECL chemiluminescence solution (BL520A, Biosharp, China) and imaged with a chemiluminescence detection system (Tano 5200Multi).

### Co-culture of CAF and PTC cell lines

The co-culture of CAF and TPC1/K1 cells was conducted using a Transwell system (with a membrane pore size of 0.4 μm). shNC CAF cells or shCD36 CAF cells suspensions were added to the Transwell inserts at a density of 2 × 10^5 cells per well in a six-well plate. The Transwell inserts were then placed into six-well plates that had been pre-seeded with TPC1 or K1 cells. The shared culture medium was prepared by mixing CAF culture medium and PTC cell culture medium at a 1:1 ratio. 1 ml of the shared cell culture medium was added to the Transwell inserts, and 2 ml of the shared cell culture medium was added to the wells of the six-well plate. For the control group, no cells were seeded in the upper chamber. The cells were cultured in a 37 °C, 5% CO_2_ incubator for 48 h. TPC1/K1 cells in the lower chamber were then collected for subsequent experiments following trypsin digestion.

### EdU assay

Cell proliferation was assessed utilizing the EdU incorporation method, facilitated by the EdU kit (Cat. No. K1075, ApexBio, USA). TPC1 or K1 cells were plated at a density of 3 × 104 cells per well in 24-well plates and allowed to adhere overnight at 37 °C. Subsequently, cells were treated with 10µM EDU for a 5-hour incubation period. Following this, cells underwent fixation with 4% paraformaldehyde for 15 min and were then treated with 0.3% Triton X-100 for membrane permeabilization for an additional 15 min. The Click Reaction Mixture was then applied to the cells for 30 min in darkness at room temperature, before being stained with Hoechst 33,342 for DNA visualization for 15 min. Fluorescence microscopy at 100× magnification was used to capture images, and ImageJ software facilitated the quantification of proliferating cells.

### Wound scratch assay

To assess the migratory capabilities of cells, the scratch wound healing assay was utilized. TPC1 or K1 cells were grown to achieve 100% confluence in a six-well plate. A precise, linear scratch was then made across the layer of cells using the tip of a pipette, followed by the replacement of the culture medium. The gap created by the scratch was documented using a microscope at 200× magnification at both the initial time (0 h) and after 24 h (24 h). The average distance across the scratch was determined at these two time points using ImageJ software, and the extent of cell migration was evaluated by computing the area percentage of the wound that had healed.

### Transwell assay

The upper chamber was pre-coated with Matrix gel, which was diluted tenfold in serum-free culture medium. TPC1 or K1 cell concentrations were adjusted to around 90,000 cells/ml using a serum-free medium. The lower chamber received a medium supplemented with 10% FBS, and 300 µl of the cell suspension was then added to the upper chamber. The setup was incubated at 37 °C in a 5% CO_2_ atmosphere for 24 h to facilitate cell migration. After incubation, each well was fixed with 700 µl of ice-cold methanol at -20 °C for 30 min at room temperature. This was followed by the addition of 700 µl of crystal violet staining solution to each well for a further 30-minute incubation. Images were taken at 200× magnification, and cell quantification was carried out using ImageJ software.

### Cell apoptosis

Apoptosis in cells was evaluated using flow cytometry with the aid of the Cell Apoptosis Detection Kit from MultiSciences (Cat. No. AT105, China). Cells from the TPC1 or K1 lines were first suspended in 1× Binding Buffer, followed by the addition of 5 µl Annexin V-APC and 10 µl 7-AAD for staining. These samples were then incubated in the dark at room temperature for 5 min. The analysis was carried out using a FACS C6 flow cytometer.

### Statistical analyses

Visualization analyses were performed using R software (version 4.1.1) with a significance level set at *p* < 0.05. Student’s t-test and analysis of variance were employed to compare quantitative data, whereas the Chi-squared test was employed for categorical variables. Correlation analysis was conducted using Spearman’s correlation test.

## Results

### Correlation between fibrosis density at the tumor invasive front and LNM in PTC

A comprehensive study involving 984 patients meeting the inclusion criteria from the First Affiliated Hospital of China Medical University was undertaken. Postoperative pathological HE-stained sections underwent panoramic scanning, with three independent pathologists delineating the tumor invasive front on all HE sections. CAFs at the invasive front of the tumor were identified using QuPath software (Fig. 1D). Immunohistochemical staining for the CAF marker α-SMA demonstrated a high concordance between the fibrotic areas identified by QuPath and α-SMA-positive expression areas (Fig. 1C). Further validation through α-SMA and CK19 immunofluorescent double staining affirmed CAF distribution delineated by QuPath at the tumor invasive front (Fig. 1B).

The fibrosis density, defined as the ratio of the fibrotic area at the tumor invasive front to the total area at the tumor invasive front, was determined. This density was ranked from high to low across all HE pathological sections, with the median value serving as the cutoff. Sections with higher fibrosis density than the median were classified as high fibrosis infiltration, and those with lower fibrosis density than the median were classified as low fibrosis infiltration. Analyzing the correlation between fibrosis infiltration levels and clinical-pathological indicators revealed a significantly higher proportion of patients with high fibrosis infiltration levels experiencing LNM. Patients with bilateral PTC and those with extrathyroidal extension exhibited a higher proportion of high fibrosis infiltration. However, no significant correlation was observed between fibrosis infiltration levels and patient sex, age, focus type, T grade, M grade, clinical stage, or concurrent Hashimoto’s disease (Table 1).

**Table 1:** Correlation analysis between the degree of fibrosis and clinical characteristics

		Low fibrosis infiltration		High fibrosis infiltration		*P* value
Metastasis	No	316	53.2%	278	46.8%	0.016
	Yes	176	45.1%	214	54.9%	
Gender	Female	379	49.3%	389	50.7%	0.488
	Male	113	52.3%	103	47.7%	
Age	<55	315	50.5%	309	49.5%	0.741
	≥ 55	177	49.2%	183	50.8%	
Location anatomic site	unilateral	394	51.7%	368	48.3%	0.056
	bilateral	98	44.1%	124	55.9%	
Focus type	unifocal	334	50.9%	322	49.1%	0.457
	multifocal	158	48.2%	170	51.8%	
T grade	T1	404	50.1%	402	49.9%	0.161
	T2	46	59.0%	32	41.0%	
	T3	23	41.1%	33	58.9%	
	T4	19	43.2%	25	56.8%	
N grade	N0	316	53.2%	278	46.8%	0.016
	N1	176	45.1%	214	54.9%	
M grade	M0	492	50.0%	492	50.0%	——
	M1	0	0.0%	0	0.0%	
Clinical stage	I	439	50.9%	424	49.1%	0.214
	II	45	45.0%	55	55.0%	
	III	7	35.0%	13	65.0%	
	IV	1	100.0%	0	0.0%	
Hashimoto disease	Without	359	50.2%	356	49.8%	0.886
	With	133	49.4%	136	50.6%	
ETE	No	454	51.0%	436	49.0%	0.065
	Yes	38	40.4%	56	59.6%	

### Differential expression of CAFs between PTC tissues with LNM (LNM-PTC) and PTC tissues without LNM (non-LNM-PTC)

**Fig. 2:**
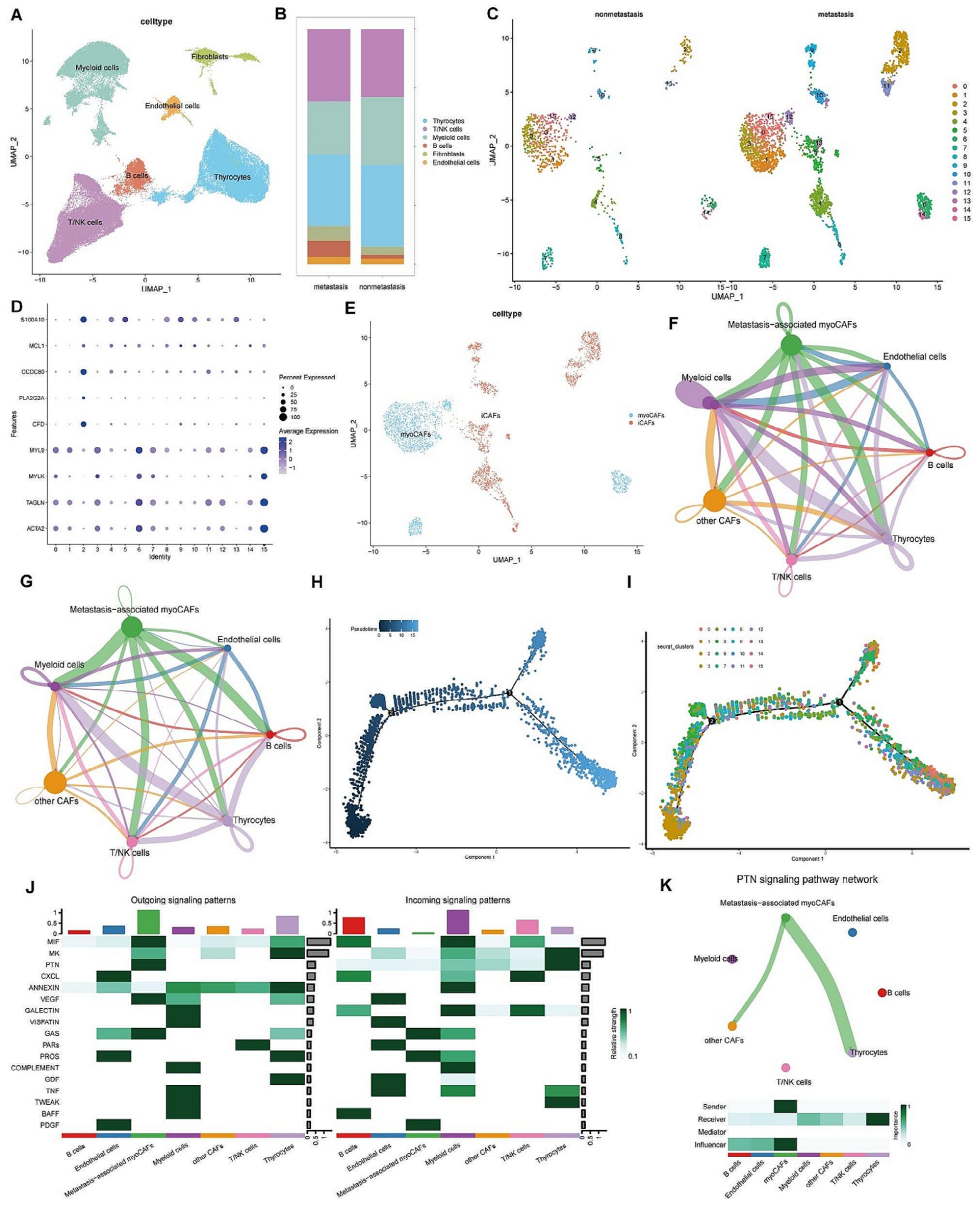
Analysis of cancer-associated fibroblasts (CAFs) in papillary thyroid carcinoma (PTC). (**A**) UMAP analysis based on cell markers revealed six distinct cell clusters within the combined GSE193581 and GSE184362 datasets. (**B**) The bar plots depict the proportional distribution of cell types in lymph node metastasis (LNM-PTC) and non-metastasis (non-LNM-PTC) PTC tissues. (**C**) Subcluster analysis and UMAP visualization of CAFs in LNM-PTC and non-LNM-PTC tissues. (**D**) Distinct marker characteristics for each CAF subcluster. (**E**) UMAP plots of myoCAFs and iCAFs in PTC tissues. (F) Cellular communication analysis (interaction strength). (**G**) Cellular communication analysis (number of interactions). (**H**-**I**) Temporal trajectory analysis of CAF clusters. (**J**) The heat map shows the incoming and outgoing signaling patterns of cell clusters. (**K**) Network visualization of the pleiotrophin (PTN) signaling pathway

In the combined GSE193581 and GSE184362 datasets, including four non-LMN (non-LNM-PTC) samples and nine PTC lesions with concurrent LNM (LNM-PTC), cell clustering based on cell markers (Figure S1A) resulted in the visualization of six distinct cell clusters through UMAP analysis. These clusters encompassed T/NK cells, myeloid cells, thyrocytes, fibroblasts, and endothelial cells (Fig. 2A). Bar plots illustrated the proportional distribution of these cell types for LNM-PTC and non-LNM-PTC (Fig. 2B). Obviously, the fibroblasts in LNM-PTC are significantly higher than that in non-LNM-PTC.

### Metastasis-associated myoCAFs strongly interacted with other cells

Subsequently, subcluster analysis and UMAP visualization of CAFs in LNM-PTC and non-LNM-PTC tissues were conducted, resulting in the subdivision of CAFs into 15 clusters (Fig. 2C), with distinct marker characteristics for each cluster highlighted in Fig. 2D. Clusters 0, 1, 3, 6, 7, 12, 14, and 15 exhibited increased MYL9, MYLK, TAGLN, and ACTA2 expression, denoted as inflammatory CAF (iCAF), whereas clusters 2, 4, 5, 8, 9, 10, 11, and 13 exhibited increased expression of S100A10, MCL1, CCDC80, PLA2G2A, and CFD, identified as myoCAF (Fig. 2E).

The subpopulation of myoCAFs that is increased in proportion in LNM-PTC tissues compared to non-LNM-PTC tissues is defined as metastasis-associated myoCAFs. Subsequently, metastasis-associated myoCAFs were extracted and annotated from LNM-PTC tissues. Cellular communication analysis revealed that these myoCAFs exhibited a higher number of communication interactions with other cells (Fig. 2F) and exhibited stronger communication intensities (Fig. 2G).

Temporal trajectory analysis of the 15 CAF clusters revealed a differentiation pattern from cluster 2 toward clusters 6, 7, 14, and 15, indicating a transition from iCAF to myoCAF, potentially associated with late-stage metastasis (Fig. 2H-I).

Signal pathway analysis of intercellular communication revealed that among all cells, metastasis-associated myoCAFs exhibited the highest output signal count. In particular, only metastasis-associated myoCAFs were identified as output signal cells in the pleiotrophin (PTN) pathway, with thyrocytes being the top recipients of this pathway (Fig. 2J). Furthermore, network visualization of the PTN signaling pathway validated similar results (Fig. 2K, Figure S1B). These results reveal that metastasis-associated myoCAFs may influence cancer cells via the PTN signaling pathway.

### Identification of the myoCAF module using hdWGCNA

**Fig. 3:**
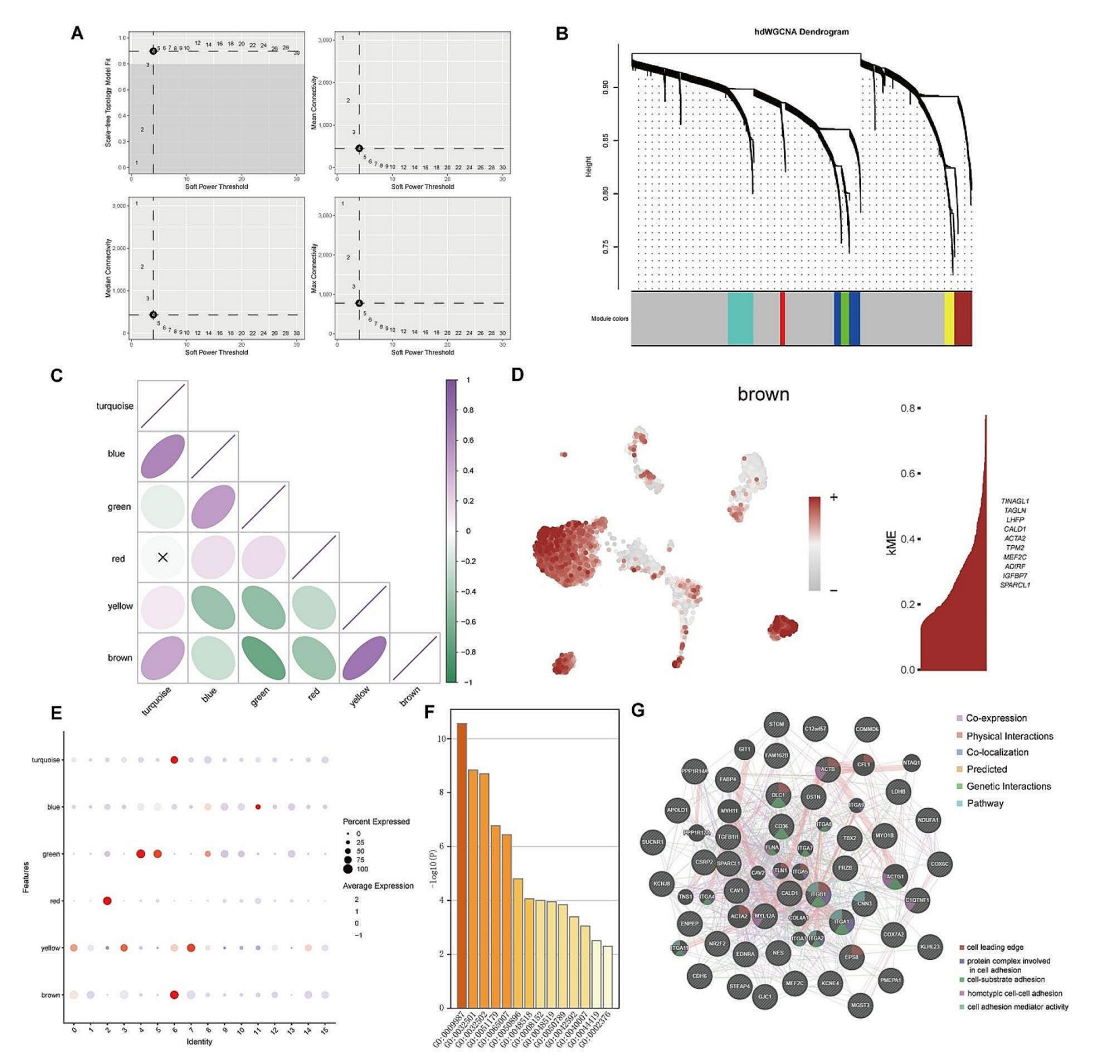
hdWGCNA analysis. (**A**) The inflection point threshold β = 4 was considered for hdWGCNA analysis. (**B**) hdWGCNA was used to analyze CAF modules, followed by the identification of six WGCNA modules. (**C**) Correlation analysis among the modules. (**D**) Mapping and high-expressing genes of brown module. (**E**) Bubble chart of the correlation between various CAF subclusters and WGCNA modules. (**F**) Gene ontology enrichment analysis of 43 genes associated with metastasis. (**G**) Protein–protein interaction network of the 43 genes associated with metastasis

In the hdWGCNA analysis, we selected a threshold at the inflection point β = 4 (Fig. 3A). The application of hdWGCNA for CAF module analysis identified six WGCNA modules—turquoise, blue, green, red, yellow, and brown (Fig. 3B). The brown module, specifically, exhibited high consistency with metastasis-associated myoCAFs, showcasing elevated expression of genes such as TINAGL1, TAGLN, LHFP, CALD1, and ACTA2 (Fig. 3D). Additional details, including mapping and highly expressing genes from other modules, are presented in Figure S1D-E. The bubble chart depicting the correlation between various CAF clusters and WGCNA modules is displayed in Fig. 3E. Notably, the brown module, especially clusters 0, 1, 3, 6, 7, 12, 14, and 15, exhibited higher proportions and expression. Correlation analysis among modules revealed a positive correlation between the brown module and the turquoise and yellow modules (associated with myoCAFs), whereas negative correlations were observed with the blue, green, and red modules (associated with iCAFs) (Fig. 3C). Subsequently, univariate Cox analysis focused on the top 100 highly expressed genes in the brown module, identifying 43 genes associated with metastasis. The results of the GO enrichment analysis for these 43 genes are depicted in Fig. 3F. The PPI network of these genes is demonstrated in Fig. 3G, indicating involvement in processes such as the cell leading edge, protein complex involved in cell adhesion, cell-substrate adhesion, homotypic cell-cell adhesion, and cell adhesion mediator activity. Pseudo-time trajectory analysis of these 43 genes revealed predominantly high expression in later stages (Figure S1F).

### Development and validation of a metastasis diagnostic model based on LNM-associated myoCAF genes

**Fig. 4:**
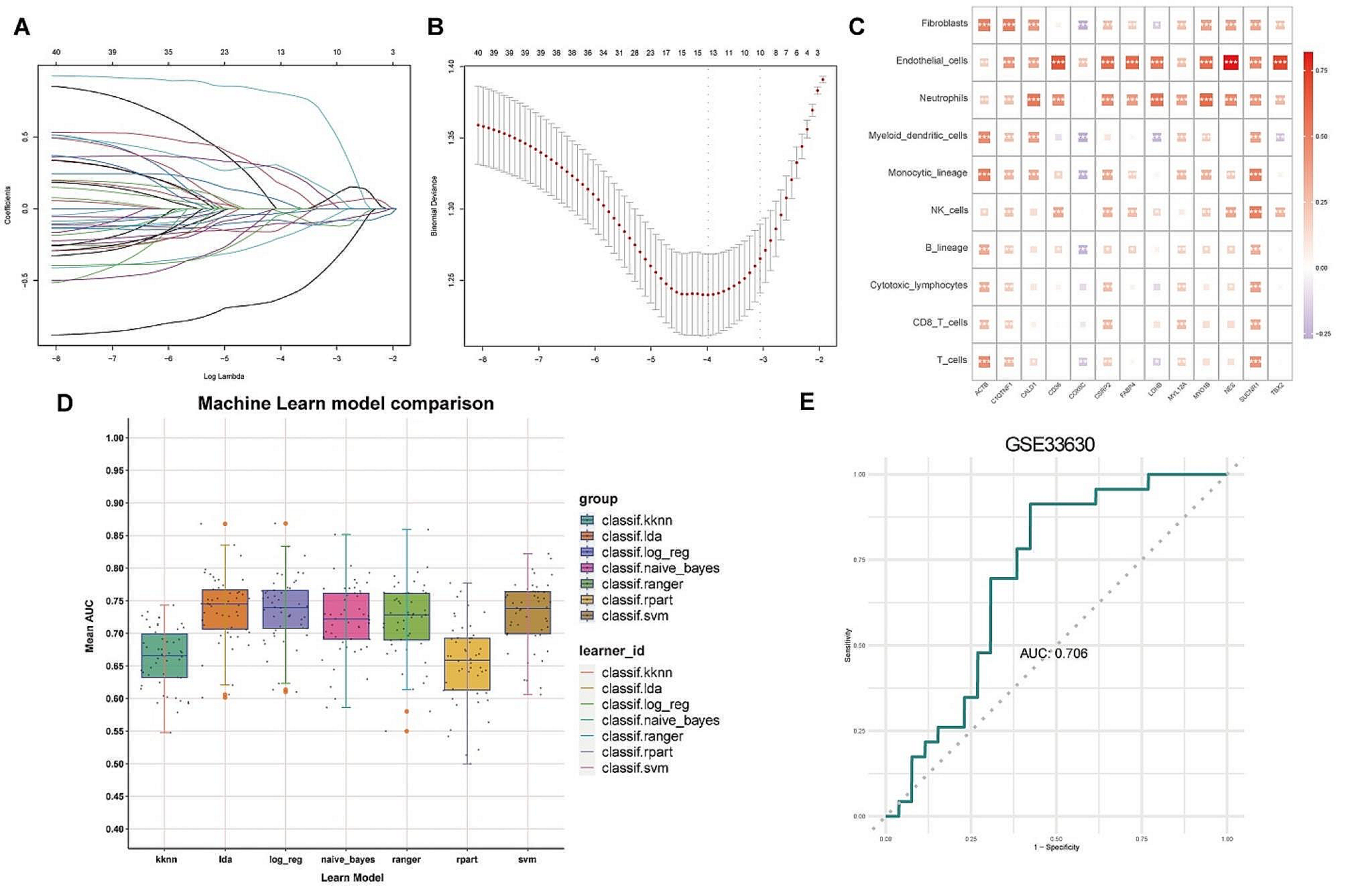
Establishment and refinement of the diagnostic model. (**A**) Least absolute shrinkage and selection operator (LASSO) coefficient profiles. (**B**) The tuning parameter (lambda) in the LASSO model was selected by performing a 10-fold cross-validation using the minimum criteria approach. (**C**) Correlation analysis of 13 metastasis-associated myoCAF-related genes with immune cells. (**D**) Area under the curve of seven deep learning models in the training set of The Cancer Genome Atlas. (**E**) Receiver operating characteristic curve of the prognostic model optimized using the support vector machine algorithm applied to the external validation set GSE33630

We constructed a diagnostic model using LASSO regression for the 43 LNM-associated myoCAF-related genes (Fig. 4A-B). A subset of 13 genes was identified for diagnosing LNM in PTC, and their relationship with stromal cells is depicted in Fig. 4C. Notably, besides COX8C and LDHB, which were significantly negatively correlated with CAFs, the other genes demonstrated significant positive correlations. To refine the diagnostic model, we used seven deep learning methods. Ultimately, we selected the SVM method to construct the final diagnostic model, which exhibited an area under the curve (AUC) of 0.706 in the external validation dataset GSE33630 (Fig. 4E).

### Identification of cancer subtypes and immune infiltration characteristics in patients with PTC

**Fig. 5:**
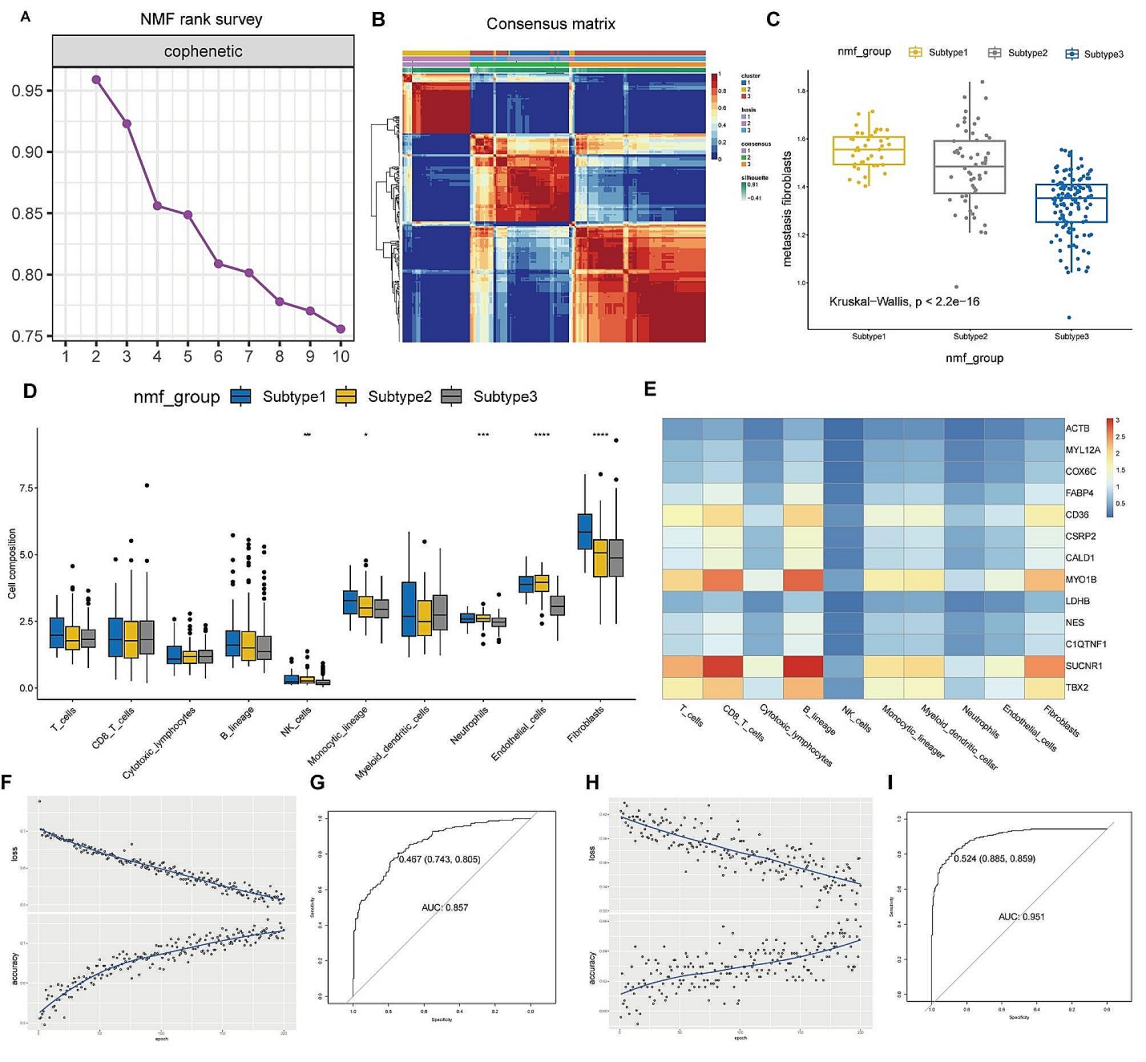
Subgroup analysis and immune infiltration characteristics in patients with papillary thyroid carcinoma (PTC). (**A**) Non-negative matrix factorization analysis stratified patients with PTC from The Cancer Genome Atlas into three distinct subgroups. (**B**) Consensus clustering outcomes showed well-defined boundaries among these subgroups. (**C**) Correlation analysis of metastasis-associated CAF infiltration across the three subgroups. (**D**) Immune cell infiltration analysis of the three subgroups. (**E**) The heatmaps show the correlation between 13 metastasis-associated myoCAF-related genes and stromal cells for each patient, followed by machine learning applied to heatmaps of all patients. (**F**-**I**) The utilization of deep learning significantly boosted the diagnostic accuracy of the model to 0.951

NMF analysis categorized patients with PTC in TCGA into three subgroups (Fig. 5A, Figure S1G), and consensus clustering results indicated distinct boundaries among these subgroups (Fig. 5B). Correlation analysis of metastasis-associated CAF infiltration levels across these three subgroups revealed that subgroup 1 exhibited the highest level of CAF infiltration, whereas subgroup 3 displayed the lowest CAF infiltration (Fig. 5C). Analysis of immune cell infiltration levels suggested that the expression of NK cells, monocytic lineage, neutrophils, endothelial cells, and fibroblasts among the three subgroups were significantly different (Fig. 5D). We generated heatmaps demonstrating the correlation of 13 metastasis-associated myoCAF-related genes and stromal cells in each patient. Deep learning was then performed for the heatmaps for all patient heatmaps (Fig. 5E). Deep learning significantly enhanced the diagnostic efficacy of the model to 0.951 (Fig. 5F-I).

### In vitro experiments confirm the pro-oncogenic role of CD36^+^CAF in PTC cell line

**Fig. 6:**
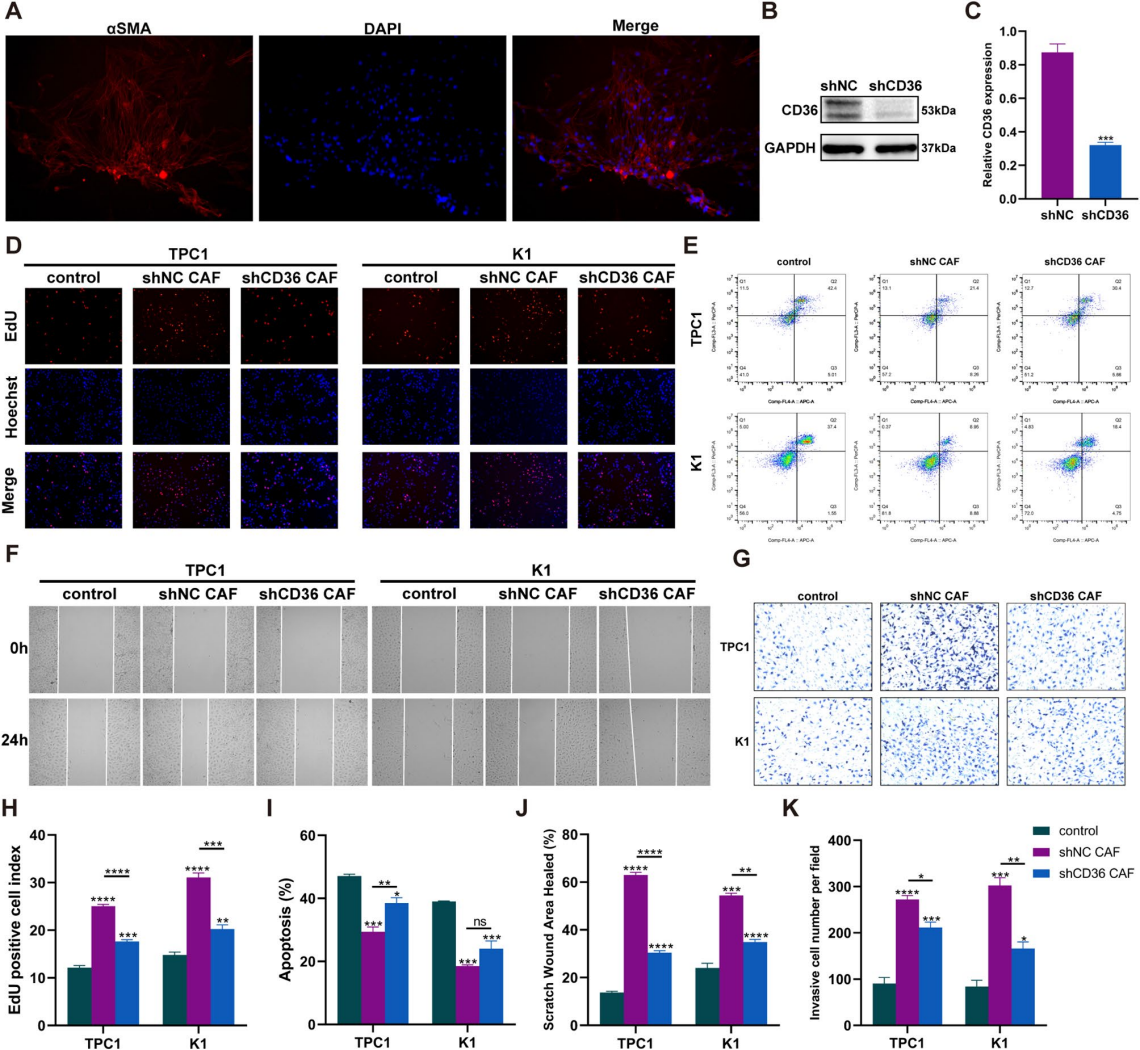
In vitro experiments confirm the pro-oncogenic role of CD36^+^CAF in PTC cell line. (**A**) Immunofluorescence staining of α-SMA in CAFs. (**B**) WB analysis of CD36 protein expression in CAF after transfecting shCD36 or control lentivirus vector and (**C**) Statistical graphs for each group. WB images are cropped. (**D**) EdU staining results and (H) statistical graphs for each group. (**F**) Scratch assays results and (**J**) statistical graphs for each group. (**G**) Transwell assays results and (**K**) statistical graphs for each group. (**E**) Flow cytometry analysis of apoptosis levels in each group and (**I**) statistical graphs. Asterisks indicate statistical comparison with the control group unless indicated otherwise on the graphs. * *p* < 0.05, ** *p* < 0.01, *** *p* < 0.001, ****p < 0.0001

Previous studies have shown that the expression of CD36 in CAFs varies across different types of cancer. Our analysis of single-cell data revealed a significant increase in CD36 expression in CAFs within PTC (Figure S1C). Primary CAF cells expressing α-SMA were isolated from PTC tissues (Fig. 6A). Subsequently, CD36 knockdown CAFs (shCD36 CAF) were successfully established through lentivirus transfection (Fig. 6B-C). Further, a Transwell co-culture system was established to investigate the effects of the control group (Transwell inserts without cell seeding), shNC CAF (Transwell inserts seeded with CAF transfected with control lentivirus vector), and shCD36 CAF (Transwell inserts seeded with CAF transfected with shCD36 lentivirus) on PTC cell proliferation, migration, invasion, and apoptosis. The results showed that the proliferation (Fig. 6D, H), migration (Fig. 6F, J), and invasion abilities (Fig. 6G, K) of TPC1 and K1 cells co-cultured with shNC CAF were significantly higher than those co-cultured with shCD36 CAF and the control group cells, and the apoptosis rate (Fig. 6E, I) was significantly lower than that in TPC1 co-cultured with shCD36 CAF and the control group cells. The proliferation (Fig. 6D, H), migration (Fig. 6F, J), and invasion abilities (Fig. 6G, K) of TPC1 and K1 cells co-cultured with shCD36 CAF were significantly higher than those of the control group cells, and the apoptosis rate (Fig. 6E, I) was significantly lower than that in the control group cells.

## Discussion

Patients with PTC and concurrent LNM face elevated mortality and recurrence risks compared to those without LNM [29]. Timely detection of LNM is crucial for reducing recurrence rates and improving the prognosis of PTC, ultimately enhancing cancer survival rates. Although nearly 80% of patients with PTC experience microscopic LNM, its identification typically relies on postoperative histopathological diagnosis [30]. Current methods, especially preoperative ultrasound examination, exhibit only 48.3% accuracy in diagnosing lymph node metastasis [31]. Consequently, establishing a reliable predictive model for diagnosing LNM in PTC, applicable in preoperative biopsies or intraoperative pathological examinations, holds paramount significance for early tumor diagnosis and preventing rapid PTC progression.

This study delves into the pivotal role of CAFs in PTC with LNM. Utilizing postoperative pathological HE slides from an extensive patient cohort, we meticulously delineated the tumor invasive front, a critical region within 1000 μm inside the tumor edge. Our findings revealed a significant correlation between high fibrosis density at the tumor invasive front and LNM. We employed a diverse range of methodologies, leading to the development of a robust diagnostic model predicting LNM in PTC based on metastasis-associated myoCAF genes. Our initial analysis, comparing CAF expression between PTC tissues with and without LNM (LNM-PTC and non-LNM-PTC, respectively), indicated a higher proportion of CAFs in LNM-PTC tissues, suggesting their potential involvement in metastatic processes. Subsequent subcluster analysis and UMAP visualization stratified CAFs into 15 clusters, revealing distinct marker profiles for myoCAFs and iCAFs. Notably, myoCAFs exhibited intense interactions with other cells, implying their significant contribution in shaping the TME. Temporal trajectory analysis unveiled a differentiation pattern from iCAFs to myoCAFs, possibly correlating with late-stage metastasis. Applying the hdWGCNA approach, we identified the brown module highly correlated with metastasis-associated myoCAFs, highlighting genes such as TINAGL1, TAGLN, LHFP, CALD1, and ACTA2. This module exhibited strong associations with metastasis-related processes, shedding light on the molecular underlying mechanisms of myoCAFs in LNM-PTC. Leveraging these findings, we constructed a diagnostic model using machine learning methods for the 43 LNM-associated myoCAF-related genes. This model exhibited remarkable accuracy, showcasing its potential in identifying PTC patients at risk of LNM. Additionally, our study included cancer subtype identification and immune infiltration analysis. NMF analysis classified TCGA patients with PTC into three subgroups, each displaying distinct immune infiltration patterns. The refined diagnostic model further enhanced its efficacy to an impressive 0.951, emphasizing its valuable clinical application potential. In summary, our study demonstrates that in PTC, CAFs infiltrate at high levels at the tumor invasive front, particularly the myoCAF subtype, which promotes PTC progression and increases the likelihood of lymph node metastasis. The diagnostic model based on myoCAF-related genes, developed using deep learning methods, can efficiently predict lymph node metastasis in PTC.

Research on CAFs in PTC is currently limited. A study of 125 PTC samples reported the use of immunohistochemistry to analyze four CAF marker proteins (FAP, α-SMA, vimentin, and PDGFR-α) and correlated them with clinicopathological features [32]. Elevated FAP and α-SMA immunoreactivity scores were associated with unfavorable tumor features, such as BRAF mutation, extrathyroidal invasion, and LNM. Dadafarin et al. proposed that MEG3 expression in tumor CAFs might drive PTC invasiveness and LNM, suggesting a potential therapeutic target [33]. Research including single-cell sequencing [34] revealed high fibroblast infiltration in PTC and their interactions with various cell types, uncovering differentially expressed fibroblast-related genes in THCA tissues. The fibrosis score model emerged as an independent prognostic factor for patients with THCA, with low fibrosis scores correlating with improved overall survival. High CAF scores were linked to aggressive phenotypes, genetic mutations, oncogenic signaling pathways, and alterations in the immune landscape alterations [34, 35]. We identified 43 metastasis-associated myoCAF-related genes, suggesting a close association with cell adhesion-related signaling pathways. We selected 13 genes (ACTB, C1QTNF1, CALD1, CD36, COX6C, CSRP2, FABP4, LDHB, MYL12A, MYO1B, NES, SUCNR1, and TBX2) to establish an LNM prediction model, achieving an optimized AUC of 0.951 via deep learning. However, further exploration and validation are warranted to understand the underlying molecular mechanisms and potential therapeutic targets.

myoCAFs are typically induced by TGF-β1 or SMAD signaling, leading to changes in cellular cytoskeleton and contributing to the formation of the ECM that promotes metastasis [36, 37]. MyoCAFs express α-SMA and secrete collagen-rich ECM [38, 39]. In various solid tumors, including esophageal [40], breast [41], colorectal [42], gastric [43], and prostate [44] cancers, myoCAFs govern malignancy-associated tumor features and are linked to poor prognosis. This study extensively explored the predictive role and underlying mechanisms of CAFs in PTC LNM, with a particular focus on myoCAFs and their strong correlation with PTC metastasis.

CD36 is a scavenger receptor expressed in various cell types, mediating lipid metabolism, immune recognition, inflammation, molecular adhesion, and apoptosis [45]. The lipid metabolism reprogramming driven by CD36 and the functional suppression of tumor-associated immune cells lead to tumor immune tolerance and cancer progression [46]. The uptake of palmitic acid (PA) by CD36 has been shown to induce phosphorylation of AKT in gastric cancer cells, inhibit the degradation of GSK3β/β-catenin, and promote gastric cancer metastasis [47]. Studies have found that in hepatocellular carcinoma (HCC), CD36^+^ CAFs exhibit high levels of lipid metabolism and expression of macrophage migration inhibitory factors [48]. In this study, CD36 positive expression in CAFs can significantly promote the proliferation, migration, and invasion abilities of PTC cells, while inhibiting the apoptosis of PTC cells. These results imply that CD36^+^CAF plays a promoting role in PTC, aligning with conclusions drawn in HCC research, thus underscoring its potential as a therapeutic target. CD36 plays a crucial role in lipid uptake, immune recognition, inflammation, molecular adhesion, and apoptosis, impacting the initiation, development, and progression of cancer. Currently, several anti-tumor drugs targeting CD36 have entered clinical trials [49]. Nonetheless, the precise mechanisms underlying CD36^+^CAF promoting role in PTC warrant further investigation.

The present is a single-center study. This is the limitation of this study. To mitigate the limitations of a single-center study, we expanded our sample size. Furthermore, the First Hospital of China Medical University is a leading hospital in Northeast China, attracting patients from across the region. This somewhat compensates for the single-center limitation by including a diverse patient population from a broad geographic area.

This study has certain limitations. The data utilized in this study originates from a singular institution, potentially constraining the generalizability of the findings. Despite efforts to increase the sample size and incorporate a varied patient demographic, future research endeavors will involve multi-center studies to improve the transferability of the results to diverse populations. Furthermore, the molecular mechanisms of CD36^+^CAFs in the progression of PTC have not been thoroughly investigated. The present study predominantly confirms their function through in vitro experiments. Subsequently, we aim to pursue comprehensive mechanistic investigations and in vivo experiments to establish a more robust scientific foundation.

## Conclusion

In conclusion, in the present study, we addressed the critical issue of the risk of LNM in patients with PTC. The analysis of postoperative HE-stained pathological slides from several patients revealed that high fibrosis density at the tumor invasive front was significantly correlated to LNM. Further, we comprehensively analyzed CAF infiltration in PTC by integrating scRNA-seq data from GSE193581 and GSE184362 datasets. Notably, we identified metastasis-associated myoCAFs exhibiting strong intercellular interactions and established a diagnostic model validated using deep learning. Next, NMF clustering revealed distinct PTC subtypes and immune infiltrate variations. In vitro experimental results indicate that CD36 positive expression in CAFs plays a promoting role in the progression of PTC. Overall, these findings provide crucial insights into the function of CAF subset in PTC metastasis.

### Electronic supplementary material

Below is the link to the electronic supplementary material.

Supplementary Material 1

Supplementary Material 2

Supplementary Material 3

## Data Availability

To protect study participant privacy, the data utilized to substantiate the conclusions of this research can be obtained by reaching out to the corresponding author upon inquiry.

## Abbreviations

THCA: Thyroid cancer
PTC: Papillary thyroid carcinoma
LNM: Lymph node metastasis
CAFs: Cancer-associated fibroblasts
HE: Hematoxylin–eosin
scRNA: seq Single-cell RNA sequencing
HdWGCNA: High dimensional weighted gene co-expression network analysis
NMF: Non-negative matrix factorization
TME: Tumor microenvironment
WSI: Whole slide imaging
TCGA: The Cancer Genome Atlas
α-SMA: Smooth muscle alpha-actin
LASSO: Least absolute shrinkage and selection operator
ROC: Receiver operating characteristic
NMF: Non-negative matrix factorization
MCP: Counter microenvironment cell population count
