# Supplementary material for: Pathomics and single-cell analysis of papillary thyroid carcinoma reveal the pro-metastatic influence of cancer-associated fibroblasts
Source: BMC Cancer. 2024 Jun 10;24:710. doi: 10.1186/s12885-024-12459-4 (PMC11163752; doi:10.1186/s12885-024-12459-4)
Supplement: Supplementary file 2 — Supplementary Material 2 [file 12885_2024_12459_MOESM2_ESM.docx]

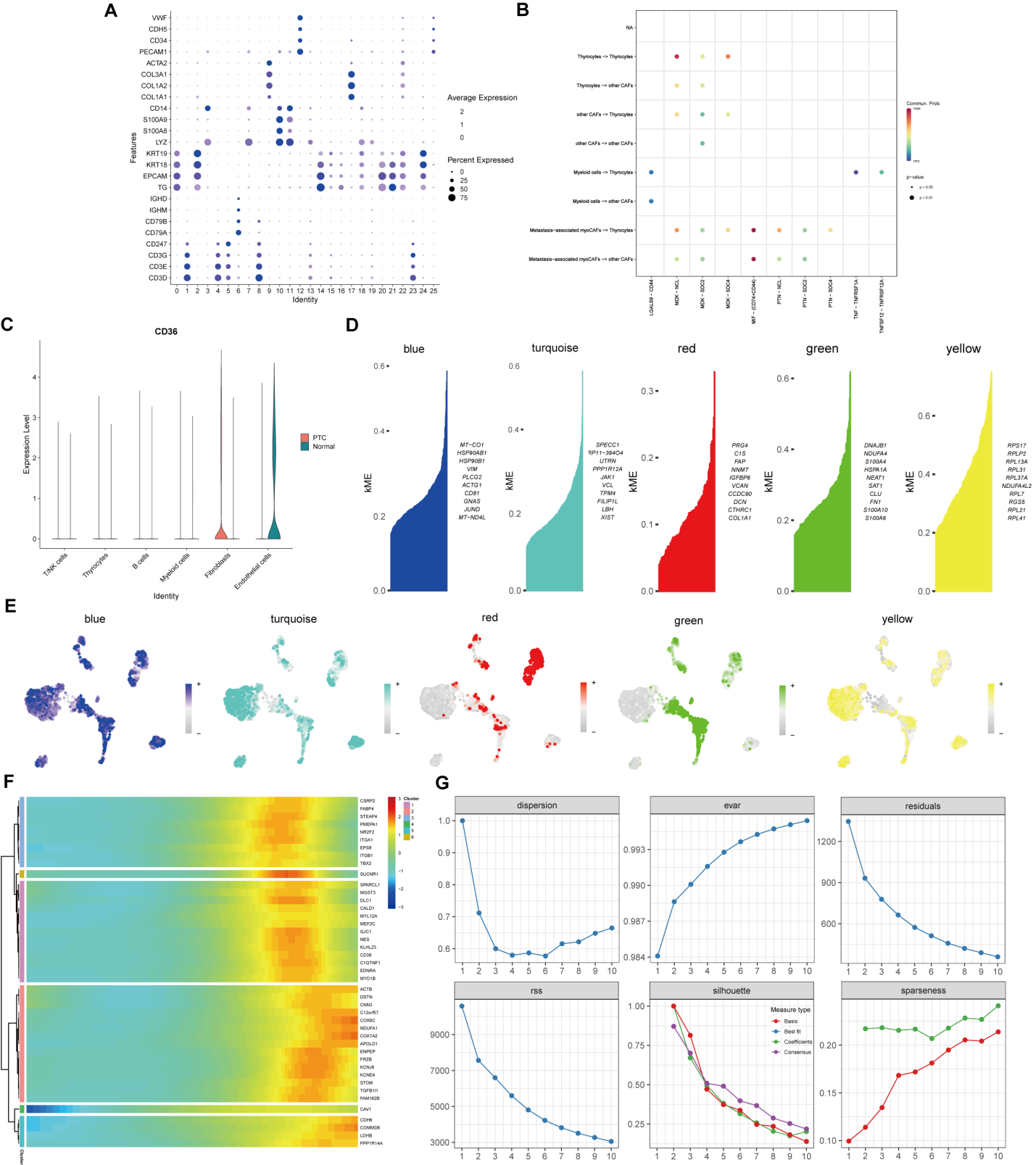


Figure S1: (A) Markers for cell subpopulation annotation. (B) Schematic diagram of intercellular communication pathways. (C) Expression of CD36 in PTC and Normal tissue.(D) Mapping of modules: blue, turquoise, red, green, and yellow. (E) High-expressing genes within modules: blue, turquoise, red, green, and yellow. (F) Pseudo-time analysis of 43 prognostic-related genes. (G) non-negative matrix factorization analysis.

Table 1: Analysis of the correlation between fibrosis infiltration levels at the tumor invasive front and clinical pathological indicators in patients with papillary thyroid carcinoma

Supplementary table 1: Statistics of Clinical Characteristics
