# Supplementary material for: Pathomics and single-cell analysis of papillary thyroid carcinoma reveal the pro-metastatic influence of cancer-associated fibroblasts
Source: BMC Cancer. 2024 Jun 10;24:710. doi: 10.1186/s12885-024-12459-4 (PMC11163752; doi:10.1186/s12885-024-12459-4)

## Slide 1
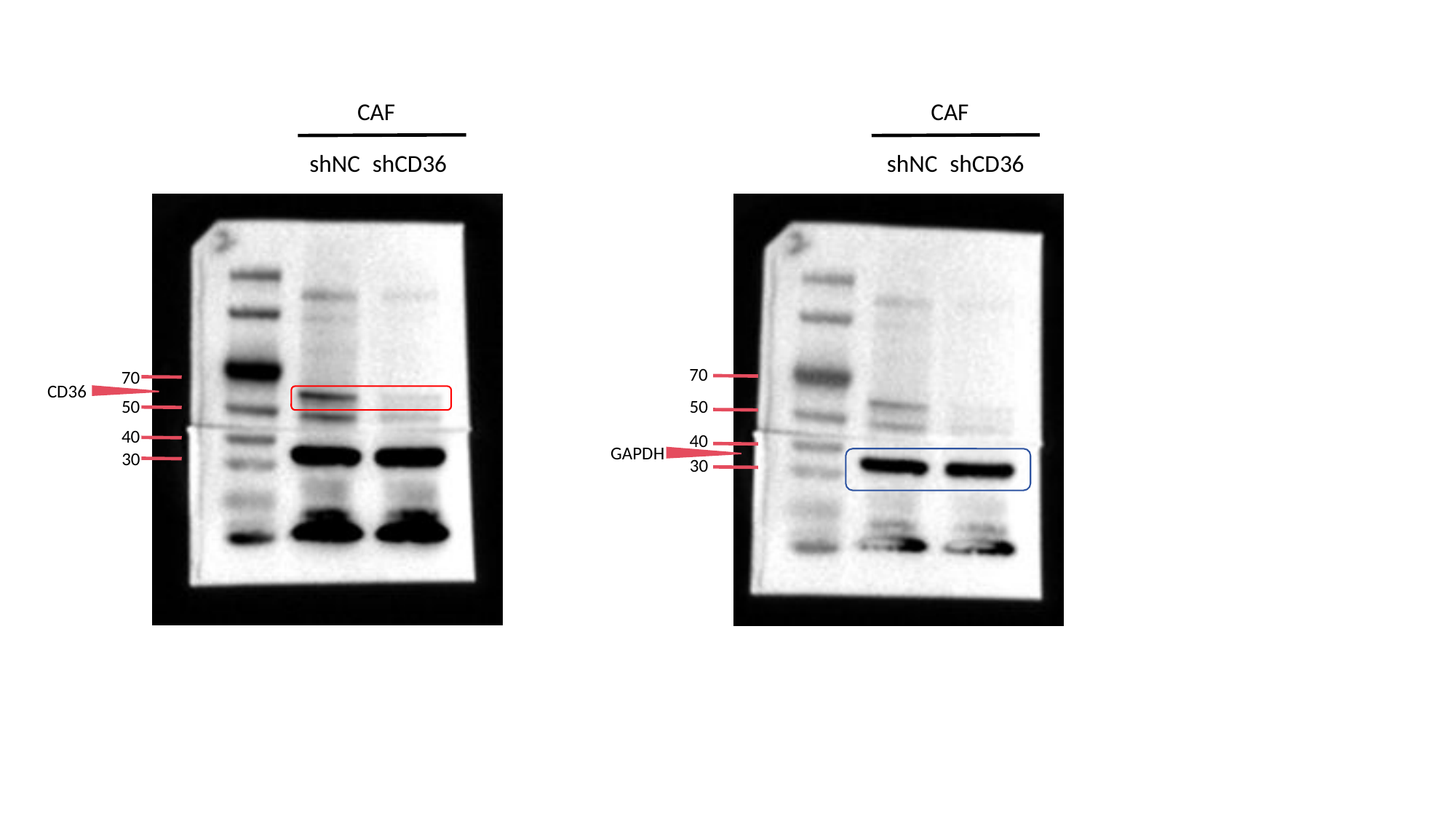

CAF
CAF
shNC
shCD36
shNC
shCD36
70
50
40
30
70
50
40
30
CD36
GAPDH

## Slide 2
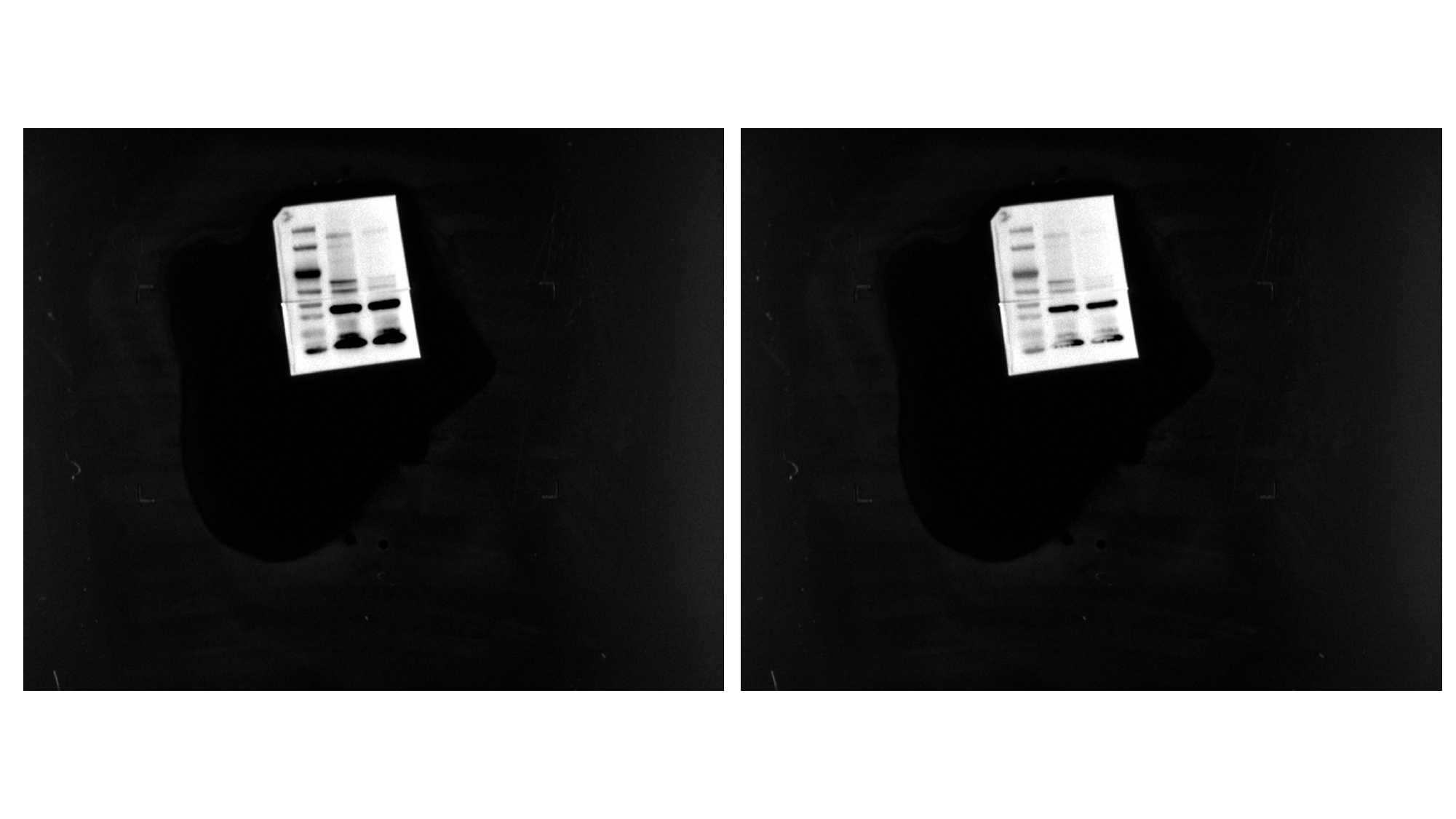

## Slide 3
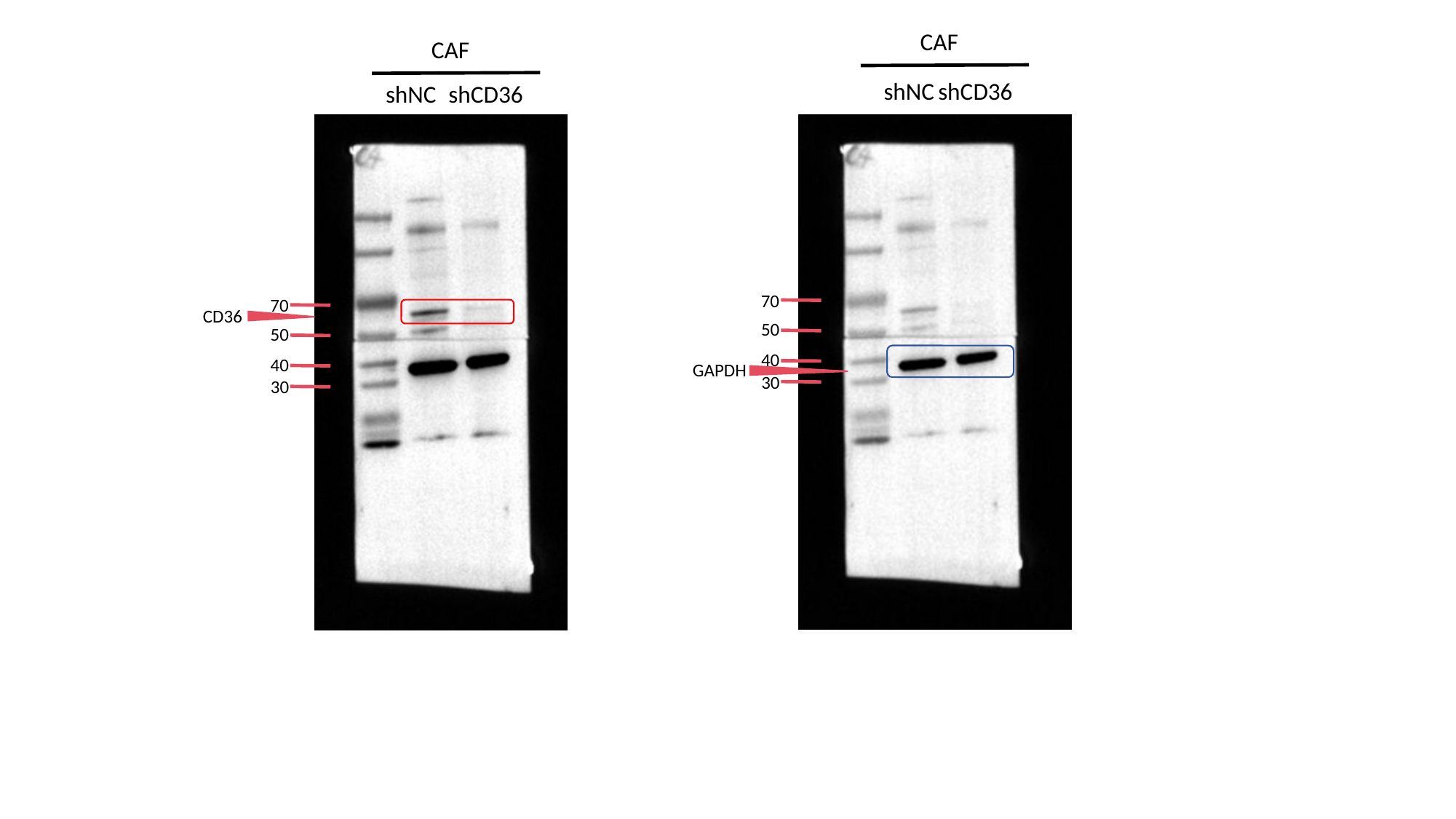

CAF
shNC
shCD36
70
50
40
30
CAF
shNC
shCD36
70
50
40
30
CD36
GAPDH

## Slide 4
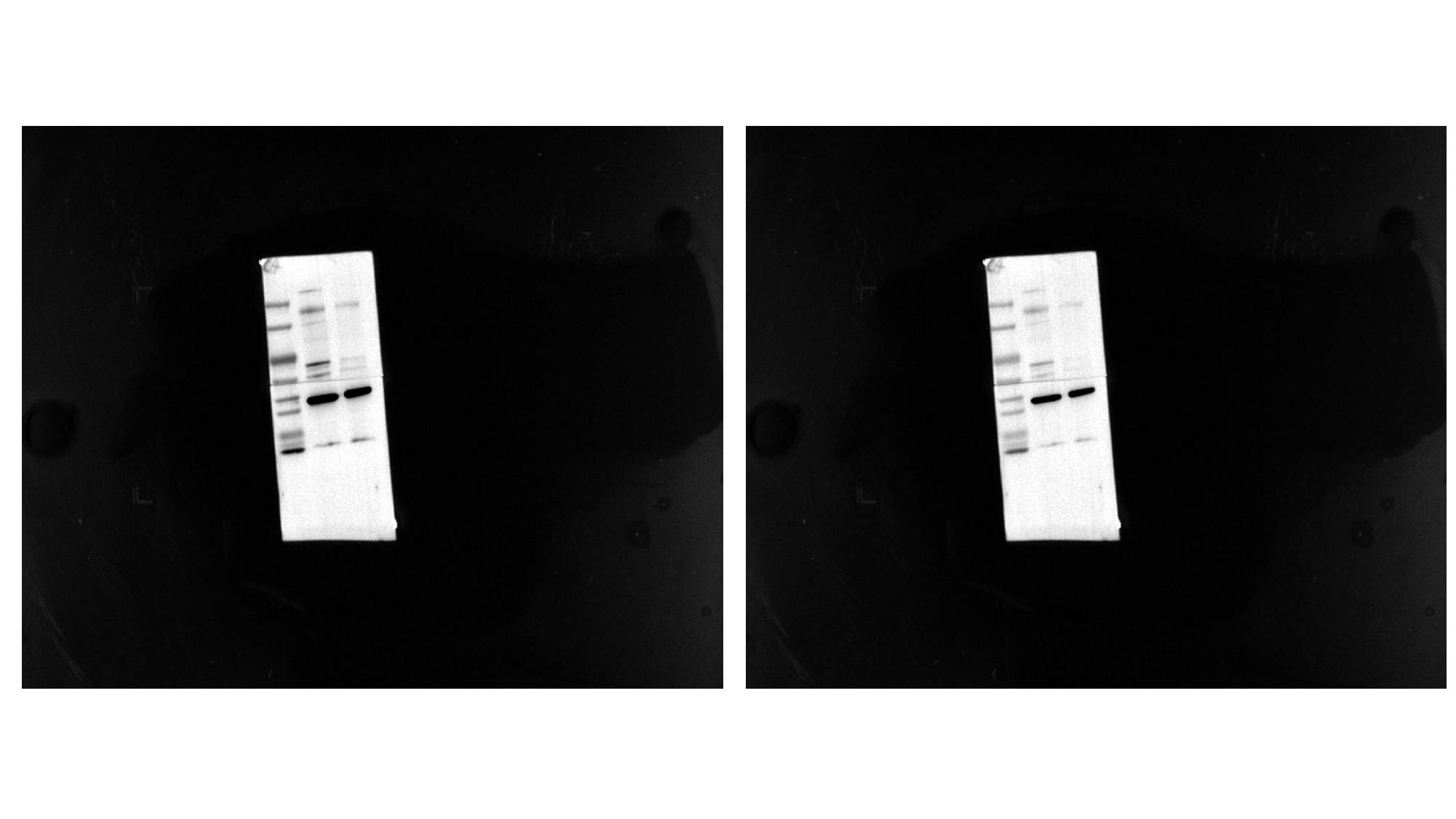

## Slide 5
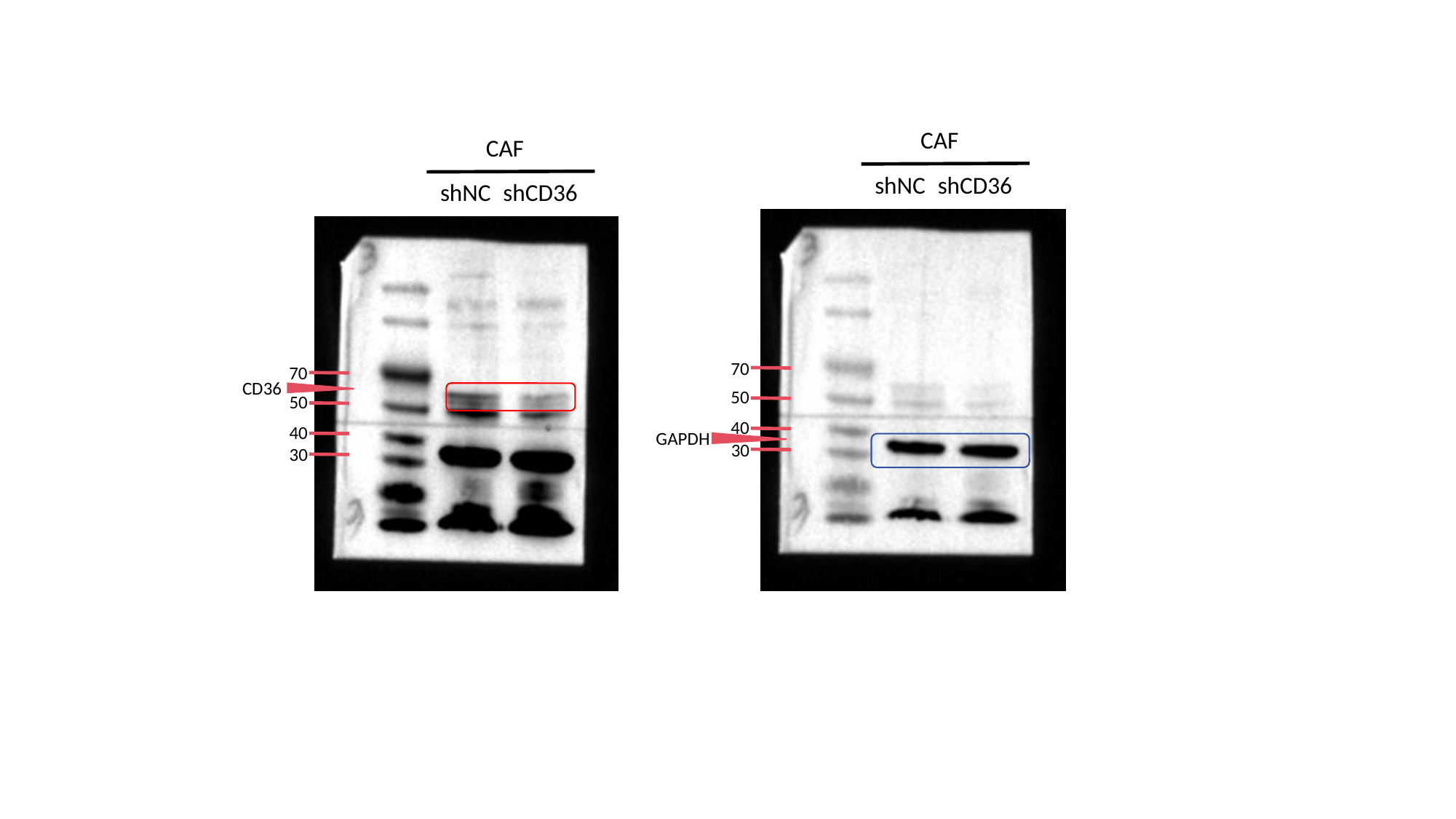

CAF
CAF
shNC
shCD36
shNC
shCD36
70
50
40
30
70
50
40
30
CD36
GAPDH

## Slide 6
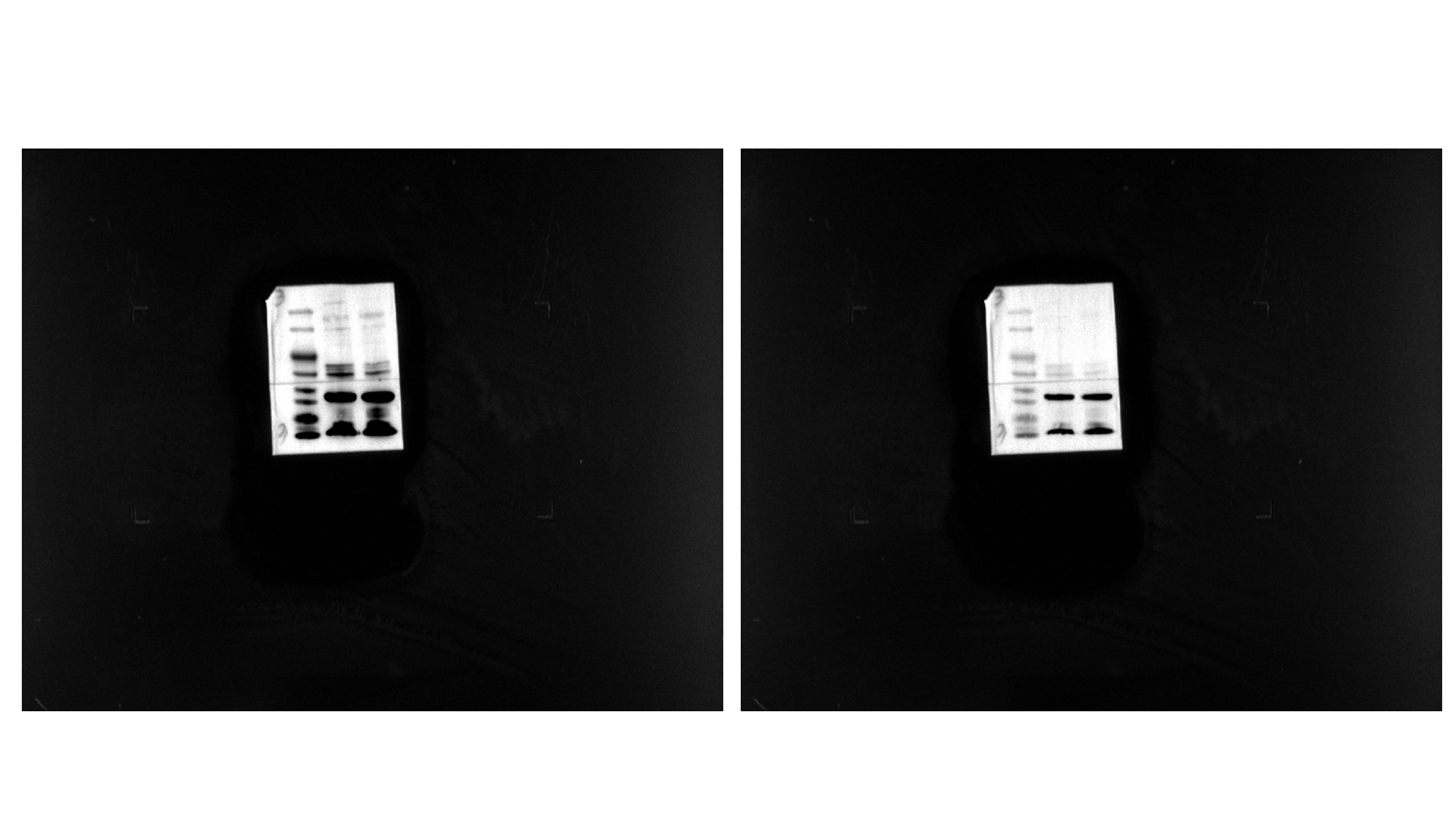

Supplement: Supplementary file 3 — Supplementary Material 3 [file 12885_2024_12459_MOESM3_ESM.pptx]
